# Facilitators and Barriers to Implementing Sustainability in Oral Health Care

**DOI:** 10.1016/j.identj.2022.08.002

**Published:** 2022-09-15

**Authors:** Catherine Minke Charlotte Volgenant, Sierou Bras, Ilona Francisca Persoon

**Affiliations:** a Department of Preventive Dentistry, Academic Centre for Dentistry Amsterdam (ACTA), University of Amsterdam and Vrije Universiteit Amsterdam, Amsterdam, the Netherlands; b Department of Cariology, Academic Centre for Dentistry Amsterdam (ACTA), University of Amsterdam and Vrije Universiteit Amsterdam, Amsterdam, the Netherlands; c Athena Institute, Faculty of Science, Vrije Universiteit Amsterdam, Amsterdam, the Netherlands

**Keywords:** Dentistry, Implementation science, Infection control, Planetary health, Sustainability

## Abstract

**Objective:**

The aim of this research was to study the facilitators and barriers to implementation of sustainable oral health care in Dutch dental practices using a qualitative research design.

**Methods:**

A conceptual framework was developed and based on 2 theories for implementation in dental practices. The framework covered 4 levels: structural, dental practice, oral health care practitioner, and method and product level. Semi-structured interviews were conducted to collect guided and in-depth data. Fourteen key stakeholders were interviewed: dentists, dental hygienists, dental assistants, managers and owners of dental practices, and suppliers of dental goods. Data were analysed using both a thematic analysis approach and open coding.

**Results:**

Participants were aware of the compromised planetary health and, in part, of their contribution to it. However, turning this awareness into action proved to be challenging. Barriers that were identified included limited knowledge and awareness of the largest sources of planetary burden in oral health care. Also, information and availability of sustainable products and methods cannot yet meet the requirements of current performance standards, costs, and infection control guidelines. Facilitators that were observed included a growing awareness to contribute to planetary health and to implement sustainability outside oral health care, especially in women and younger people. Overviews and guides of existing sustainable methods are available, but additional methods and products should be developed as well.

**Conclusions:**

Many participants considered infection control guidelines as the most prominent barrier to sustainable oral health care. Women felt more involved with planetary health compared to men, which is in line with the concept of ecofeminism. It is essential for stakeholders to collaborate to reach the next levels of implementation. Action is required on all levels to secure both oral and planetary health. Now is the time to act.

## Introduction

Health care workers strive to protect and promote their patients’ health. The environment severely impacts this health. Climate change caused by unsustainable human behaviour can directly increase mortality of all living beings by means of extreme weather conditions.^1^ Indirectly, climate change and depletion of resources lead to socioeconomic and health inequality.^2^ Additionally, increasing global temperatures have been linked to a rise in vector-borne infectious diseases.^1^^,^^3^ More than 10 years ago, Lafferty^4^ predicted a rise in infectious diseases resulting from climate change. The effects of climate change have been observed to contribute to the morbidity and mortality of the COVID-19 pandemic.^5^^,^^6^ Considering the great impact of the environment on health, health care workers should contribute to the global effort to promote planetary health.^7^

Oral health care contributes to the effect of global health care on planetary health. It contributes to approximately 3% of the carbon footprint of British health care.^8^ Duane et al^9^ reported that 65% of carbon dioxide emissions in the oral health care sector originate from transport. Decreasing this emission can be achieved by reducing the number of appointments through providing preventive care and combining appointments for families.^9^ The filling material amalgam can release mercury, which is toxic to both human beings and the environment.^10^ Hence, it is essential to restrict its use and to attend to proper waste disposal.^11^ Furthermore, dental practices produce large amounts of waste composed of mixed materials due to the use of single-use items.^12^^,^^13^ This is partly because of requirements to prevent cross-transmission of infectious diseases. An audit regarding the waste of dental practices in Australia revealed that roughly 90% of the produced waste resulted from products used to comply to infection control guidelines.^14^

Duane et al^15^ have provided several suggestions for embedding and implementing sustainability in oral health care. However, little is known about experiences of oral health care practitioners with implementing these sustainable initiatives. Even less is known about why these initiatives have or have not been implemented. Grose et al^16^ suggested that infection control guidelines were experienced as a barrier to working ecologically and sustainably in dental practice. Nevertheless, these guidelines provide recommendations essential to providing safe care and should not be disregarded. Martin et al systemically reviewed the literature on implementation of sustainable practices in dentistry.^17^ They observed several barriers to implementation, including a lack of awareness and knowledge, carbon emissions from commuting, and lack of optimal reuse of dental materials and biomedical waste. Concurrently, these barriers were identified as opportunities to facilitate implementation.^18^

Several of these factors might affect the implementation of sustainability in Dutch dental practices. The aim of this study is to explore facilitators and barriers to implementing ecologically sustainable methods in oral health care at different levels and stages. Qualitative research using interviews provides a deeper understanding of the motivations and perceptions of the relevant stakeholders who are involved in providing oral health care.

## Methods

Semi-structured interviews with relevant stakeholders were conducted to get an in-depth understanding of their insights, perceptions, and activities.^19^ A stakeholder analysis identified several relevant parties including day-to-day oral health care practitioners, such as dentists, dental hygienists, and dental assistants. Practice owners and managers also significantly influence daily activities and applied products. Distributors of dental supplies and services were also identified as relevant stakeholders, as they have an overview of the available sustainable products and current demands from practices. Participants were purposively sampled aiming at maximum diversity. Initially, the sample lacked older men and practice owners. Snowball sampling was used to include them as participants in the study. Eligible participants were contacted by email with information about the study and to invite them for the interview. Potential participants received an additional information letter. Participants received a small reward in the form of a bar of Fair Trade and CO_2_-compensated chocolate.

The field of social sciences typically studies implementation using a theoretical framework to map the various processes and aspects necessary for this implementation in a specific setting. Therefore, existing knowledge on barriers and facilitators to implementation in dental practices can be used.^20^^,^^21^ Chaudoir et al identified a framework reflecting the different levels at which implementation takes place: incidental innovation, factors on a patient or provider level, factors on an organisational level, and factors on a structural level.^20^ Additionally, it is crucial to study the implementation of innovations in dental practices. Simpson^21^ provides a comprehensive theory in which the specific stages of implementation are described: training, adoption, implementation, and practice improvement. Training refers to learning about an innovation, whereas adoption covers the process of acceptance of an innovation. Finally, after actual implementation has taken place, practice improvement is used to evaluate the change.^21^

A theoretical framework was devised, based on the 2 aforementioned theories^20^^,^^21^ (Figure). The framework was used to compose a topic list with relevant themes to discuss during the interviews (see Appendix A). Semi-structured interviews were conducted to collect guided and in-depth data using an interview guide, but at the same time the interviewer was able to elaborate on topics. This allowed the interviewer to explore new topics with the participants.^22^ Throughout the interviews, implementation was discussed starting from the structural level and gradually moving to lower levels. The various stages of implementation were discussed for each of these levels. The interview guide was piloted with 3 students and 1 dentist, other than the participants. Interviews were conducted by a female researcher experienced in dentistry and qualitative research (SB), who was in her twenties and conscious of planetary health without having yet implemented strong sustainability measures in her everyday life. The interviews were conducted in Dutch from April to June 2020. The COVID-19 pandemic impeded face-to-face contact; therefore, the interviews were performed by audio or video calls whilst the participants were at home or in another private environment. Member checks were performed by sending the respective participants a summary of the main findings from their own interview to control for possible deviations of the researcher's interpretation of the interview.^23^ The interviews were audio recorded and transcribed verbatim, after which the audio files were discarded.

**Fig:**
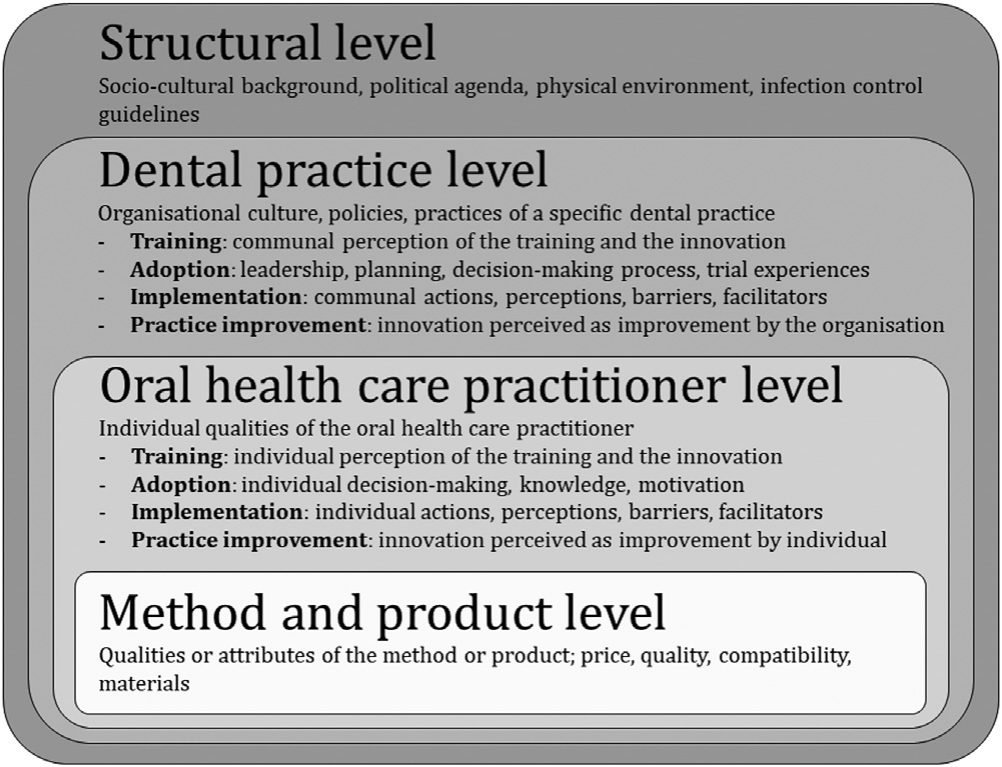
Conceptual framework for implementation of sustainable oral health care. This framework is constructed based on the framework for implementation of health innovations^20^ and the framework for implementing sustainable oral health promotion interventions.^21^

Transcripts were analysed using ATLAS.ti (version 8; Scientific Software Development GmbH). Transcripts were processed on the basis of a thematic analysis.^24^ Data analysis included both deductive and inductive coding. Text fragments were coded based on the framework and interview guides. Using open coding, additional codes were derived from the data to explore factors that did not fit into the current conceptual framework.^25^ One researcher (SB) analysed and coded the first 2 transcripts and constructed a coding frame. The codes and the coding frame were discussed with a male researcher experienced in qualitative research (EU). The coding frame was supplemented and finalised during coding of the remaining transcripts (SB). Themes were also discussed with 2 researchers experienced in dentistry (CMCV, IFP). Recurring themes were observed amongst different stakeholders, and no new themes were observed despite the limited number of stakeholders being interviewed, thereby affirming data saturation.^26^

This study is reported in accordance with the Standards for Reporting Qualitative Research^27^ (see Appendix B). The study protocol was approved by the Internal Review Board of the Academic Centre for Dentistry Amsterdam (reference number 202059) and the study was exempted from the Dutch Medical Research Involving Human Subjects Act. Written informed consent was obtained from all participants prior to data collection. Transcripts were numbered and included no data that could be linked directly to the participant. Only the researcher who collected the data had access to a file that could link the anonymised transcripts to respective participants.

## Results

Interviews were held with 14 stakeholders from various locations throughout the Netherlands (Table). Interviews generally lasted 60 minutes (range, 25–90 minutes).

**Table:** Demographics of the participants in this study.

Number	Sex	Age (y)	Role	Area	Practice/company
1	Female	55	Dentist	Village	Practice A
2	Female	32	Dental assistant	Village	Practice A
3	Female	29	Dentist	Town	Practice B
4	Female	27	Dentist	City	Practice C/D
5	Female	63	Dental assistant	City	Practice E
6	Female	33	Dentist	City	Practice F
7	Male	54	Product category manager dental	Town	Company A
8	Male	60	Quality manager dental	Town	Company A
9	Male	52	Senior sales and export manager	Town	Company B
10	Male	37	Dentist	Town	Practice G
11	Male	61	Dentist and co-owner of a dental practice	Town	Practice H
12	Male	26	Dental hygienist and dentistry student	Town	Practice I
13	Male	21	Dental assistant	Village	Practice A
14	Female	38	Dental hygienist and practice manager	Village	Practice J

### Structural level

All participants agreed that little information was available regarding sustainable methods in oral health care in professional literature or in dental education. Also, 3 participants^1^^,^^3^^,^^6^ considered education programmes and professional literature as reliable and suitable sources for this type of information. Two participants^3^^,^^6^ indicated having unsuccessfully searched for sustainable dental products online.

All participants agreed that infection control guidelines can impede working sustainably in oral health care. They mentioned mandatory single-use disposables, such as gloves and masks. Also, 7 participants^1^^,^^3^^,^^4^^,^^5^^,^^10^^,^^12^^,^^14^ acknowledged that they would like to separate waste in their practice. However, the bulk of this waste is potentially contaminated. Participants indicated that infection control guidelines prohibit recycling of contaminated waste to prevent cross-transmission.

*“One would like to separate the waste used for the patients, which is an enormous volume, the majority of all waste in the practice. But I don't think, from a hygiene perspective, that that's possible.”—Participant 1*

Additionally, 6 participants^1^^,^^3^^,^^4^^,^^6^^,^^10^^,^^11^ stated that they were interested in using more sustainable products and methods in their practice but that they experienced a lack of available options. However, the participants working for dental suppliers^7^, ^8^, ^9^ indicated that interest in sustainable dental products is limited and, consequently, that production and supply would increase if the demand would increase. This vicious circle delays progress towards a sustainable future in oral health care.

*“It's like the story of the chicken and the egg. If there is no demand, it will not be produced and if it's not produced, there will be no demand. So, at some point someone will have to take the first step.”—Participant 11*

### Dental practice level

Several participants mentioned leadership and the decision-making process on this level. Three dentists^1^^,^^3^^,^^4^ indicated that their position as an employed dentist made it difficult for them to implement sustainable changes. Two participants with management positions in their practice^11^^,^^14^ indicated that they were able to make the desired sustainable changes. This emphasises that awareness and willingness of management is essential for implementation of sustainable practices.

Oral health care practitioners mentioned reduction of energy use as the most frequently taken sustainable measure within their practice. These measures included thermal insulation of the building, using LED lighting, or providing rooms with sensors instead of manual light switches. Five participants^5^^,^^6^^,^^10^^,^^11^^,^^14^ would like to use solar panels to power the practice. However, this was experienced as difficult to implement if they were not the owner of the practice or the building.

A change of methods is perceived as more difficult compared to switching from one consumable product to the next by 4 participants.^1^^,^^2^^,^^13^^,^^14^ New methods could require training or a large investment, whereas this is typically not the case for switching consumable products. Therefore, implementation of new methods at a practice level can act as barriers to change.

*“I said: o, then we just need to get another automated washer disinfector. And then he said: uh, do you have any idea how expensive that is?”—Participant 3*

### Oral health care practitioner level

Nine participants^1^, ^2^, ^3^, ^4^, ^5^, ^6^^,^^10^^,^^11^^,^^14^ mentioned that starting and keeping the debate on sustainability is important for successful implementation. Raising awareness and applying simple sustainable practices was perceived as easier in smaller dental practices. Eight participants^1^, ^2^, ^3^, ^4^, ^5^, ^6^^,^^9^^,^^10^^,^^14^ considered their colleagues to not feel the need to work more sustainably. This perception of being the only person to value sustainability could act as a barrier to taking action.

*“Nobody else was talking about it except for me and the wife of the dental practice owner and then they started taking it more seriously. So, you need several people to make a change.”—Participant 3*

The 7 female participants were more likely to value sustainability highly and take more sustainable actions, both at work and at home, compared to the male participants. Specifically, 3 female dentists^3^^,^^4^^,^^6^ indicated that they had already taken many actions towards sustainability in their private lives.

*“Well, I'm vegan now. So, I don't eat meat or dairy and we separate our waste now, at least I think we do, but we probably don't always, and I try not to fly [in an airplane] if I don't have to.”—Participant 4*

### Method and product level

Several participants^3^^,^^4^^,^^10^ questioned whether all products advertised as sustainable actually were sustainable throughout the total life cycle of the product. They considered it difficult to select sustainable products, as many factors should be taken into account and not all necessary information is available.

Four participants^3^^,^^11^^,^^13^^,^^14^ had experience using sustainable products when providing patient care. They indicated that some products were more expensive or performed below standard. Seven participants^1^, ^2^, ^3^, ^4^, ^5^^,^^10^^,^^13^ mentioned the possibility of tips for the high-volume evacuator or for the air water syringe that can be decontaminated and reused. Both products are considered to be a good alternative compared to plastic disposables. However, 4 participants^3^^,^^11^^,^^13^^,^^14^ mentioned certain shortcomings of these reusable items with regards to their performance or hygiene.

The most important attribute of the described product was its price; a dental practice is a business that needs to be run. Unfortunately, sustainable products are generally more expensive compared to the standard product. Therefore, the price can act as a barrier towards the use of sustainable products.

*“[The product] has to offer the same level of convenience compared to the materials we currently use and preferably at almost the same price. A small raise is permissible, but within limits of course.”—Participant 11*

## Discussion

This study found a predominantly positive attitude towards sustainability in oral health care. Also, the results highlighted the importance of collaboration in implementing sustainable change; only if oral health care practitioners, managers, practice owners, producers, and suppliers work together can significant changes be made towards a sustainable future.

A previous study concluded that professionals are mainly focussed on their professional activities and are less engaged in sustainability as compared to general society.^17^ Similar to our study, other research has also found that health care workers who act sustainably in their private lives are more likely to be motivated to act sustainably at work as well.^16^^,^^28^ A study amongst Dutch oral health care practitioners identified similar results with regards to motivation towards implementing sustainable practices.^13^ Several studies stress the importance of collaboration amongst all stakeholders, including early adopters, the government, and oral health care professional organisations.^13^^,^^15^ Previous studies have also identified infection control guidelines as a barrier to sustainable oral health care.^13^^,^^15^^,^^16^ The importance of raising awareness for increasing sustainable actions at all levels in oral health care is emphasised by previous reports.^15^^,^^29^

Reduction of energy consumption is identified as a significant method to contribute to planetary health, both in this study and other studies.^13^^,^^15^^,^^17^^,^^29^ Although transport contributes significantly to CO_2_ emission in dentistry,^8^ restricting transport of people and goods was hardly mentioned by our participants. Another method to promote planetary health is to promote oral health and prevent disease in order to avoid invasive, specialist treatments or even treatment at all.^15^^,^^29^ Conversely, participants focussed a lot on composition of products, single-use products, and waste reduction.^9^^,^^17^ This focus on products is logical, since changing behaviour is more difficult.^30^

The COVID-19 pandemic could have shifted the attention from sustainability to hygiene. However, the current results indicate that the pandemic might have shifted the attention towards finances. Most Dutch dental practices only provided emergency treatments for 5 weeks during the first lockdown, during which the interviews for the present study were conducted.^31^ Providing only limited regular care has inevitably led to missed revenue.^32^ Because of shortages of, for instance, personal protective equipment, prices have significantly increased simultaneously.^32^ This could have led to our findings indicating that price was perceived as an important product attribute and that participants were less likely to invest in sustainability. Policies to recover from the pandemic should be aligned with measures to promote planetary health.^2^ These public health challenges can only be managed with a global effort.

All participants were motivated towards sustainable action, which contradicts a previous study by Grose et al.^29^ This could indicate that selection bias might have occurred during the recruitment process. The study population of 5 employed dentists and 1 dentist who was co-owner of a dental practice differs from the Dutch population of dentists, where approximately 50% of the dentists own a practice.^33^ This number is decreasing with the rising number of collaborative practices and commercial chains of dental practices, hence the rise of employed dentists. However, this study demonstrated that employed dentists typically have little influence on the decision-making process in the dental practice. The influence of these new types of practices may be the subject of future research.

The results indicated a higher level of motivation towards sustainability amongst female participants, which is in line with the theory of ecofeminism. This theory links the oppression of women in society to the degradation of the natural environment, which could make women more likely to care about sustainability.^34^ The theory of ecofeminism claims that women have been trying to protect the environment for decades.^35^ Currently, the majority of the dentistry students in the Netherlands are female, whereas previously most dentists were male.^33^ Feminisation of the profession could provide positive prospects for sustainability in Dutch oral health care.

## Conclusions

Participants were aware of the compromised planetary health and, in part, of their contribution to it. However, turning this awareness into action proved to be challenging. Several barriers were identified, such as limited knowledge and awareness of the largest sources of planetary burden in oral health care. Also, there is a lack of both information and availability of sustainable products and methods that meet the requirements of current performance standards, costs, and infection control guidelines. Facilitators that were observed included a growing awareness to contribute to planetary health and to implement sustainability outside oral health care, especially in women and younger people. Future research could focus on how to minimise the observed barriers and to make use of the observed facilitators. It is essential that all stakeholders collaborate and act on all levels to secure both oral and planetary health. Now is the time to act.

## Funding

The authors have received no financial support for the research, authorship, or publication of this article.

## Conflict of interest

None disclosed.

## Appendix Supplementary materials

Image, application 1

Image, application 2
