# Supplementary material for: Facilitators and Barriers to Implementing Sustainability in Oral Health Care
Source: Int Dent J. 2022 Sep 15;72(6):847–52. doi: 10.1016/j.identj.2022.08.002 (PMC9676545; doi:10.1016/j.identj.2022.08.002)
Supplement: Supplementary file 1 [file mmc1.pdf]

Topic List

(translated from Dutch)

Introduction

General information about the participant, education, educational program regarding sustainability, current job, vision on sustainability in general and regarding the oral health care practice.

Attitude towards sustainability

Personal attitude/interest in the subject, feasibility in the oral health care practice, current situation in the practice, attitude of the practice, chances for further developments, factors which make it easier / more difficult to work sustainably in the practice. Who within the team bears the most responsibility for this, who is most interested and why. What would you like to change regarding sustainability in the practice and do you think sustainability should be an important point of attention within a dental practice and why (not)?

Experience in sustainability

What have you experienced already in practices regarding sustainability? What have you applied already and why? How did you come up with this idea, how did you experience this? Recommendations for others, other interesting initiatives and barriers/facilitators.

Possible sustainable initiatives

Several examples are given of sustainable options for the oral health care practice. The participant is asked to what extent he/she is familiar with this, how this may be applied and how the participant thinks it can be improved in future.

Examples: separating waste; alternative cleaning agents; disposables & packaging materials; reduced water or energy use; transport (also of materials); sustainable consumer products.
